# Elevated Plasma Level of Interferon-*λ*1 in Chronic Spontaneous Urticaria: Upregulated Expression in CD8^+^ and Epithelial Cells and Induction of Inflammatory Cell Accumulation

**DOI:** 10.1155/2016/5032051

**Published:** 2016-06-30

**Authors:** S. F. Wang, X. Q. Gao, Y. N. Xu, D. N. Li, H. Y. Wang, S. H. He

**Affiliations:** ^1^Allergy and Clinical Immunology Research Centre, The First Affiliated Hospital of Liaoning Medical University, Jinzhou, Liaoning 121001, China; ^2^Department of Dentistry, The Second Affiliated Hospital of Liaoning Medical University, Jinzhou, Liaoning 121001, China; ^3^Allergy and Inflammation Research Institute, The Key Immunopathology Laboratory of Guangdong Province, Shantou University Medical College, Shantou 515031, China

## Abstract

Interferon- (IFN-) *λ*1 is regarded as a potent bio-active molecule in innate immunity. However, little is known about its role in chronic spontaneous urticaria (CSU). We therefore investigated expression of IFN-*λ*1 in CSU, its cellular location, and its influence on inflammatory cell accumulation by using flow cytometry analysis, skin tissue dispersion, immunohistochemical stain, and a mouse peritoneal inflammation model. The results showed that level of IFN-*λ*1 was 2.0-fold higher in plasma of the patients with CSU than the level in healthy control (HC) subjects. Among leukocytes examined, only CD8^+^ T cells expressed more IFN-*λ*1 in CSU blood. Double labeling immunohistochemical staining revealed that IFN-*λ*1^+^ inflammatory cells such as mast cells, eosinophils, B cells, neutrophils, and macrophages were mainly located in dermis, whereas epidermis tissue highly expressed IFN-*λ*1. IFN-*λ*1 induced a dose-dependent increase in number of eosinophils, lymphocytes, mast cells, macrophages, and neutrophils in the peritoneum of mice at 6 h following injection, which was inhibited by pretreatment of the animals with anti-intercellular adhesion molecule- (ICAM-) 1 and/or anti-L-selectin antibodies. In conclusion, IFN-*λ*1 is likely to play a role in the pathogenesis of CSU. Blocking IFN-*λ*1 production may help to reduce the accumulation of inflammatory cells in the involved CSU skin.

## 1. Introduction

CSU, which has no discernable external cause, comprises the majority of cases of chronic urticaria [1]. It is recognized to have an autoimmune cause including production of IgE autoantibodies against autoantigens and generation of IgG autoantibodies against Fc*ε*RI, IgE, or both, which might chronically activate mast cells and basophils [2]. These autoantibodies have been shown to activate blood basophils and cutaneous mast cells* in vitro* and induction of a cellular infiltration of CD4^+^ T lymphocytes, monocytes, neutrophils, eosinophils, and basophils [3]. However, the exact physiopathology of CSU remains unknown [4].

It was reported that cytokines may contribute to the pathogenesis of CSU. Thus, higher serum levels of the CXCL8, CXCL9, CXCL10, and CCL2 were observed in CSU patients compared to the healthy group [5]; serum levels of interleukin- (IL-) 18 [6], RANTES [7], and IL-31 [8] were significantly higher in CSU than in controls. In the dermis of lesional skin of CSU, there were increases in IL-4(+) and IL-5(+), IL-33(+), IL-25(+), and thymic stromal lymphopoietin (TSLP) (+) cells compared to nonlesional skin [9]. However, levels of interferon- (IFN-) *λ*1 (IL-29) in CSU remain uninvestigated.

IFN-*λ*1 belongs to the Type-III IFN family [10] and is categorized to the superfamily of human Class II cytokines [11]. The receptor for IFN-*λ*1 is composed of IFNLR1 and IL-10R2. IFN-*λ* exhibits several common features with type I IFNs: antiviral activity [12], antiproliferative activity, and* in vivo* antitumour activity [13]. It is also involved in immune regulatory activities, such as the regulation of T helper (h)1/Th2 responses [14], modulation of dendritic cell function [15, 16], and induction of mast cell accumulation [17]. The previous reports that levels of IFN-*λ*1 were elevated in the plasma of the patients with asthma [17] and that sputum IL-29 mRNA levels were higher in the steroid-treated asthmatic patients than in healthy controls [18] implicate that the cytokine is likely to contribute to the pathogenesis of allergic airway disorders. Based on the “allergic march” concept, we anticipate that IFN-*λ*1 may be involved in the pathogenesis of CSU. The aim of the current study is to investigate the potential role of IFN-*λ*1 in CSU, its cellular location, and its influence on inflammatory cell accumulation.

## 2. Materials and Methods

### 2.1. Reagents

The following compounds were purchased from Sigma-Aldrich (St. Louis, MO, USA): collagenase (type I), hyaluronidase (type I), lipopolysaccharides (LPS), eotaxin, alkaline phosphatase conjugated goat anti-mouse IgG, peroxidase conjugated sheep anti-rabbit IgG antibody, rabbit anti-human IFN-*λ*1 antibody, Fast Red TR/Naphthol AS-MX, and human serum albumin (HSA). Dulbecco's modified Eagle's medium (DMEM) was from Gibco BRL (Grand Island, NY, USA). Rat anti-mouse CD 62L (L-selectin) antibody and hamster anti-mouse CD54 [intercellular adhesion molecule 1 (ICAM-1)] antibody, Cytofix/Cytoperm*™* Fixation/Permeabilization Kit, rabbit anti-human CD8 antibody (anti-CD8), PE-conjugated goat anti-rabbit IgG, FITC-anti-human CD4, and APC-anti-human CD19 were purchased from BD Biosciences Pharmingen (Bedford, MA, USA). Human IL-4, IL-10, thymic stromal lymphopoietin (TSLP) enzyme-linked immunosorbent assay (ELISA) kits were purchased from R&D Systems (Minneapolis, MN). Alkaline phosphatase conjugated mouse anti-human tryptase G3, alkaline phosphatase conjugated mouse anti-human chymase B7, mouse anti-human MBP, and mouse anti-human CD20 (Clone L26) antibodies were obtained from Chemicon International Inc. (Temecula, CA, USA). Rabbit anti-human lysozyme (anti-Lys) antibody was from Abcam (Cambridge, UK). Rabbit anti-human lactoferrin antibody (anti-Lac) was from Jackson ImmunoResearch Laboratories, Inc. (West Grove, PA, USA). IFN-*γ* ELISA kit, PE/Cy7-anti-human CD8, PE/Cy7-anti-human CD14, BV421-anti-human CD16, PE/Cy7-anti-human CD123, PerCP-anti-human HLA-DR, BV421-anti-human CD117, PerCP-anti-human Fc*ε*RI*α*, and PE/Cy7-anti-human CD34 antibodies were purchased from Biolegend (San Diego, CA, USA). DAB + substrate chromogen system was purchased from Dako Cytomation (Carpinteria, CA, USA). Allergens for skin prick were supplied by ALK-Abelló, Inc. (Denmark). Human IFN-*λ*1 ELISA kit was purchased from eBioScience (Los Angeles, CA, USA). Most of the general-purpose chemicals such as salts and buffer components were of analytical grade.

### 2.2. Patients and Samples

A total of 23 CSU, 18 eczema, and 32 HC subjects were recruited in the study. The diagnosing criteria of CSU [19] and eczema [20] were conformed with the ones published previously. The informed consent from each volunteer according to the Declaration of Helsinki and agreement with the ethical committee of the First Affiliated Hospital of Liaoning Medical University or General Hospital of Shenyang Military Area Command was obtained. The general characteristics of the patients and control subjects were summarized in Table 1. Peripheral venous blood sample (10 mL) from each patient or HC subject was taken into an EDTA containing tube before centrifugation at 450 g for 10 min. The cells were used for flow cytometric analysis, and plasma was collected and frozen at −80°C until use. Specimens of human foreskin tissues were removed at circumcision from normal subjects and were used for immunohistochemistry and flow cytometric analysis. They were collected from the Department of Pathology, the First Affiliated Hospital of Liaoning Medical University. The protocol for ethical use of human tissue in research was according to the Declaration of Helsinki (2000) and approved by the Committees of the First Affiliated Hospital of Liaoning Medical University.

### 2.3. Animals, Cell Line, and Culture

BALB/c male mice (18–22 g) were obtained from Vital River Laboratory Animal Technology Co. Ltd. (Beijing, China). The animals were bred and reared under strict ethical conditions according to international recommendations. They were housed in the Animal Experimental Centre of the First Affiliated Hospital of Liaoning Medical University in a specific pathogen-free environment with free access to standard rodent chow and water, at a constant temperature of 23–28°C and relative humidity of 60–75%. The animal experiment procedures were approved by the Animal Care Committee at Liaoning Medical University.

### 2.4. Flow Cytometry Examination of IFN-*λ*1 Expression in Peripheral Blood Leukocytes

To detect IFN-*λ*1 expression on CD4^+^ T cells, CD8^+^ T cells, neutrophils (CD16^+^ polynucleated cells), eosinophils (CD16^−^ polynucleated cells) monocytes (CD14^+^ cells), B cells (CD19^+^ cells), and basophils (CD123^+^HLA-DR^−^ cells), the following antibodies were added to different testing tubes (1) to detect IFN-*λ*1 expression in basophils: PE/Cy7-anti-human CD123 and PerCP-anti-human HLA-DR; (2) to detect IFN-*λ*1 expression in CD4^+^ and CD8^+^ T cells: FITC-anti-human CD4 and PE/Cy7-anti-human CD8; (3) to detect IFN-*λ*1 expression in monocytes, B cells, neutrophils, and eosinophils: PE/Cy7-anti-human CD14, APC-anti-human CD19, and BV421-anti-human CD16 before 200 *μ*L of whole blood being added at room temperature for 15 min in dark. Following ligation of red blood cells, white blood cells were fixed and permeabilized by using Cytofix/Cytoperm Fixation/Permeabilization Kit according to the manufacturer's instructions. The cell pellets were then resuspended and rabbit anti-human IFN-*λ*1 followed by PE-conjugated goat anti-rabbit IgG antibodies were added at 4°C for 30 min. Finally, cells were resuspended in fluorescence-activated cell sorting- (FACS-) flow solution and analyzed with FACS verse flow cytometer (BD Biosciences, San Jose, CA). A total of 10,000 events were analyzed per population for each sample. Data were analyzed with CellQuest software (BD Immunocytometry systems).

### 2.5. Cell Dispersion from Skin Tissue and Flow Cytometry Analysis of IFN-*λ*1 Expression

The procedure for dispersing skin tissue cells was mainly adopted from the procedure described previously by He et al. [21]. Briefly, skin tissues were digested with 2.0 mg/mL collagenase, 1.0 mg/mL hyaluronidase, and 1 *μ*g/mL DNase in DMEM for 70 min at 37°C. After centrifugation at 450 g for 6 min, the dispersed skin tissue cells were fixed by using a Cytofix/Cytoperm solution for 20 min at 4°C. Cells were then incubated with each labeled monoclonal antibody including BV421-anti-human CD117, PerCP-anti-human Fc*ε*RI *α*, PE/Cy7-anti-human CD34, and rabbit anti-human IFN-*λ*1 (PE-conjugated goat anti-rabbit IgG) antibodies at 4°C for 30 min in the dark. Finally, cells were analyzed with FACS verse flow cytometer.

### 2.6. Immunohistochemical Stain and Cell Count

Specimens fixed in Carnoy's fixative and embedded in paraffin wax. The staining procedure for double labeling immunohistochemistry was mainly adopted from the procedure described previously by He et al. [22]. For each section, the number of positively stained cells was counted in at least 30 fields (the area of each field equals 0.19 mm^2^). Sections from six different donors were examined.

For detection of tryptase or chymase and IFN-*λ*1, sections of skin were incubated with biotin conjugated mouse anti-human IFN-*λ*1 antibody for 2 h followed by ExtrAvidin®-peroxidase conjugate for 30 min and alkaline phosphatase conjugated G3 anti-tryptase antibody or alkaline phosphatase conjugated B7 anti-chymase antibody for 2 h. For detection of CD20 (marker of B cells) and IFN-*λ*1, the sections were incubated with biotin conjugated mouse anti-human IFN-*λ*1 antibody and L26 anti-CD20 (IgG2a isotype) antibody for 2 h and followed by adding ExtrAvidin-peroxidase conjugate for 30 min, and alkaline phosphatase conjugated goat anti-mouse IgG for 1 h. For detection of IFN-*λ*1 and CD8 (marker of cytotoxic T cells), lysozyme (marker of macrophages) or lactoferrin (marker of neutrophils), the sections were incubated with biotin conjugated mouse anti-human IFN-*λ*1 and anti-CD8, anti-lysozyme or anti-lactoferrin antibodies for 2 h, respectively, and followed by adding ExtrAvidin-peroxidase conjugate for 30 min and peroxidase conjugated sheep anti-rabbit IgG antibody for 1 h. Staining was developed over 4 min using DAB chromogen system and another 4 min with Fast Red TR/Naphthol AS-MX before being counterstained with Mayer's haematoxylin and mounted in aquamount.

For staining epidermis tissue, sequential sections were incubated with biotin conjugated mouse anti-human IFN-*λ*1 antibody for 2 h followed by ExtrAvidin-peroxidase conjugate for 30 min. Color was developed by using DAB chromogen system before being counterstained with Mayer's haematoxylin and mounted in aquamount.

### 2.7. Mouse Peritoneal Injection and Cell Count

The procedure was adapted from that described previously [23]. Briefly, various concentrations of IFN-*λ*1 in the presence or absence of its specific antibody (anti-IFN-*λ*1, 3.0 *μ*g/mL), eotaxin (10 ng/mL), HSA (10 ng/mL), LPS (1.0 *μ*g/mL), and normal saline (NS) were injected in 0.5 mL volumes into the peritoneum of mice before their peritoneal lavage being collected for differential cell analysis. At 6 h following injection, animals were killed, and their peritoneal lavage fluids were collected and centrifuged. Cells were resuspended in 2.0 mL MEM, stained with 0.1% trypan blue, and enumerated using an Improved Neubauer haemocytometer (for total cell numbers). Cytocentrifuge preparations were made and stained with modified Wright's stain. Differential cell counts were performed for a minimum of 500 cells. The results were expressed as absolute numbers of lymphocytes, neutrophils and macrophages, eosinophils, and mast cells per mouse peritoneum.

For the experiments investigating cell migration mechanism, groups of mice were pretreated intravenously with monoclonal antibodies against the adhesion molecules L-selectin (anti-CD62L, 1 mg·kg^−1^) and ICAM-1 (anti-CD54, 1 mg·kg^−1^) [24], respectively, for 30 min before intraperitoneal injection of IFN-*λ*1. At 6 h following injection, mice were sacrificed and their peritoneal lavages were processed as described above. Control animals received an equivalent dose of the corresponding normal rat or hamster IgG (isotype control).

### 2.8. Determination of Levels of Cytokines

Levels of IL-4, IL-10, TSLP, IFN-*γ*, and IFN-*λ*1 in the plasma of CSU, eczema, and HC subjects were measured by using ELISA kits according to the manufacturer's instruction.

### 2.9. Statistical Analysis

Statistical analyses were performed by using SPSS software (Version 17.0, IBM Corporation). Data are displayed as a boxplot, which indicates the median, interquartile range, the largest, and smallest values for the number of experiments indicated. Plasma levels of cytokines are presented as scatter plot. Where Kruskal-Wallis analysis indicated significant differences between groups, for the preplanned comparisons of interest, the paired Mann-Whitney *U*-test was employed. Where analysis of variance indicated significant differences between groups with ANOVA, Student's *t*-test was applied. Correlations were determined by using Spearman rank correlation. For all analyses, *P* < 0.05 was taken as significant.

## 3. Results

### 3.1. Elevation of IFN-*λ*1 Levels in the Plasma of the Patients with CSU

**Figure 1:**
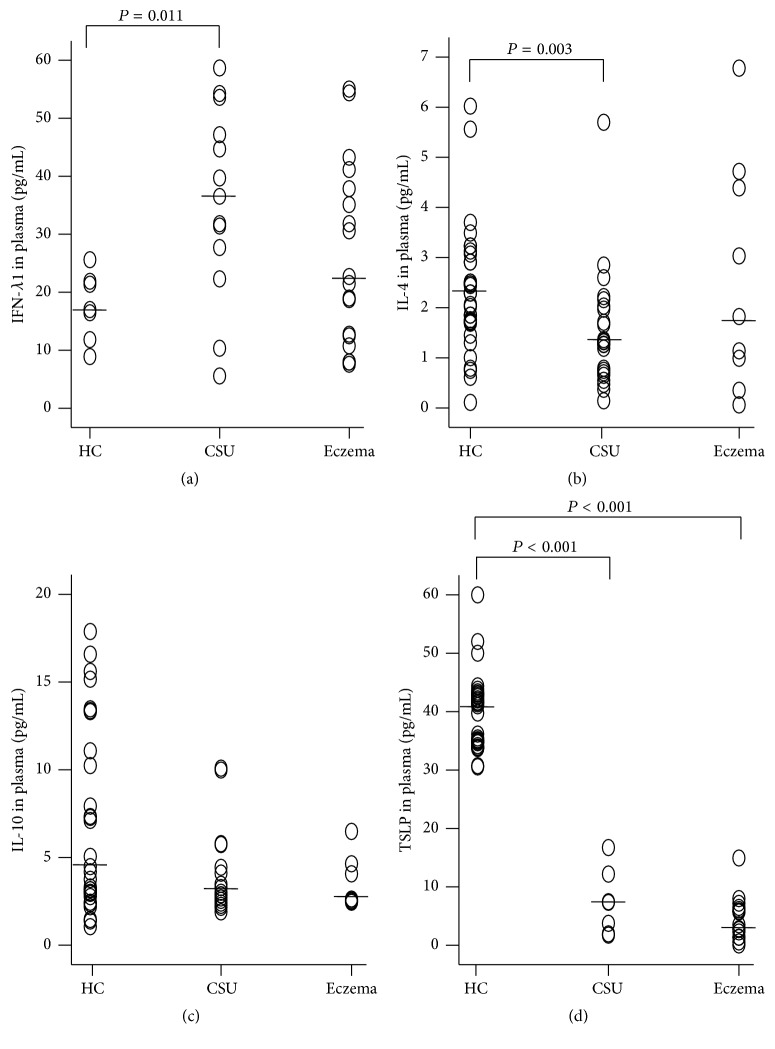
Scatter plots of the levels of interferon- (IFN-) *λ*1 (a), IL-4 (b), IL-10 (c), and thymic stromal lymphopoietin (TSLP) (d) in the plasma of the patients with urticaria and healthy control subjects (HC). Each symbol represents the value from one subject. The median value is indicated with a horizontal line. *P* < 0.05 was taken as statistically significant. See Table 2.

IFN-*λ*1 is a newly identified immune active cytokine that plays a role in antivirus [12] and some other immune regulatory activities [14]. With ELISA, we found that the level of IFN-*λ*1 was 2.0-fold higher in the plasma of the patients with CSU than the level in HC subjects (Figure 1(a)). In contrast, plasma IFN-*λ*1 level of the patients with eczema was similar to that of HC subjects (Figure 1(a)). The levels of IL-4 (Figure 1(b)) and TSLP (Figure 1(d)) in the plasma of the patients with CSU were lower than that of HC subjects. Levels of IFN-*λ*1 correlated negatively with IL-4, IL-10, and TSLP in the plasma of CSU and HC subjects (Table 2). Levels of IFN-*γ* in the plasma of CSU, eczema, and HC subjects were low and inconsistent (data not shown).

### 3.2. Enhanced Expression of IFN-*λ*1 in Peripheral Blood Leukocytes of the Patients with Urticaria

**Figure 2:**
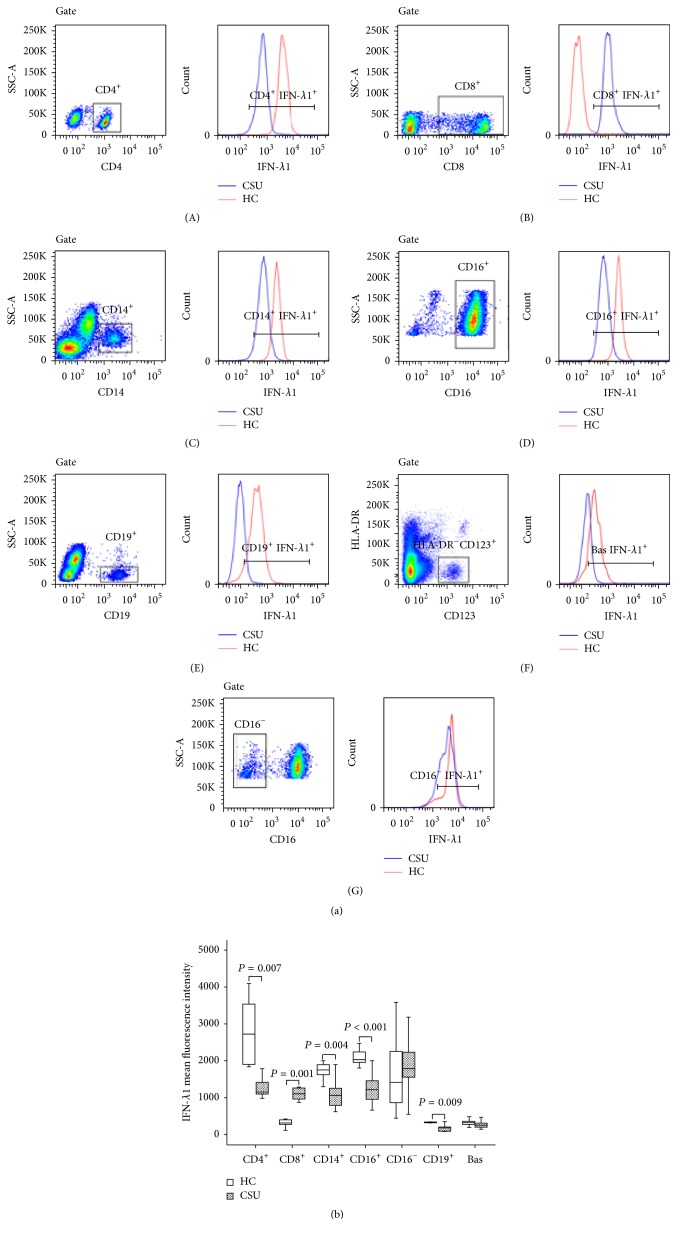
Flow cytometry analysis of the expression of interferon- (IFN-) *λ*1 in peripheral blood leukocytes. (a) showed a representative graph of the changes in the mean fluorescence intensity (MFI) of IFN-*λ*1 in CD4^+^ (helper T cells), CD8^+^ (cytotoxic T cells), CD14^+^ (monocytes), CD16^+^ (neutrophils), CD19^+^ cells (B cells), CD123^+^HLA-DR^−^ (basophils), and CD16^−^ (eosinophils) in healthy control subjects (HC) and patients with urticaria, respectively. Gate represents gating strategy. (b) represents a boxplot of the expression of IFN-*λ*1 in peripheral blood leukocytes of the patients with urticaria and HC, which indicates the median (horizontal line), interquartile range, the largest, and smallest values other than outliers (whiskers) and the outliers (O). Each piece of data represented a group of minimum 7 subjects. *P* < 0.05 was taken as statistically significant.

In order to identify the potential source of IFN-*λ*1, we investigated the expression of IFN-*λ*1 in peripheral blood leukocytes. The results showed that the mean fluorescence intensity (MFI) of IFN-*λ*1 expression was not increased in CD4^+^, CD14^+^, CD16^+^, CD19^+^, CD16^−^, and CD123^+^HLA-DR^−^ cell populations of CSU in comparison with that in HC subjects. However, IFN-*λ*1 expression in CD8^+^ cells of CSU was enhanced by 2.6-fold (Figure 2).

### 3.3. Flow Cytometric Analysis of IFN-*λ*1 Containing Cells in Skin

**Figure 3:**
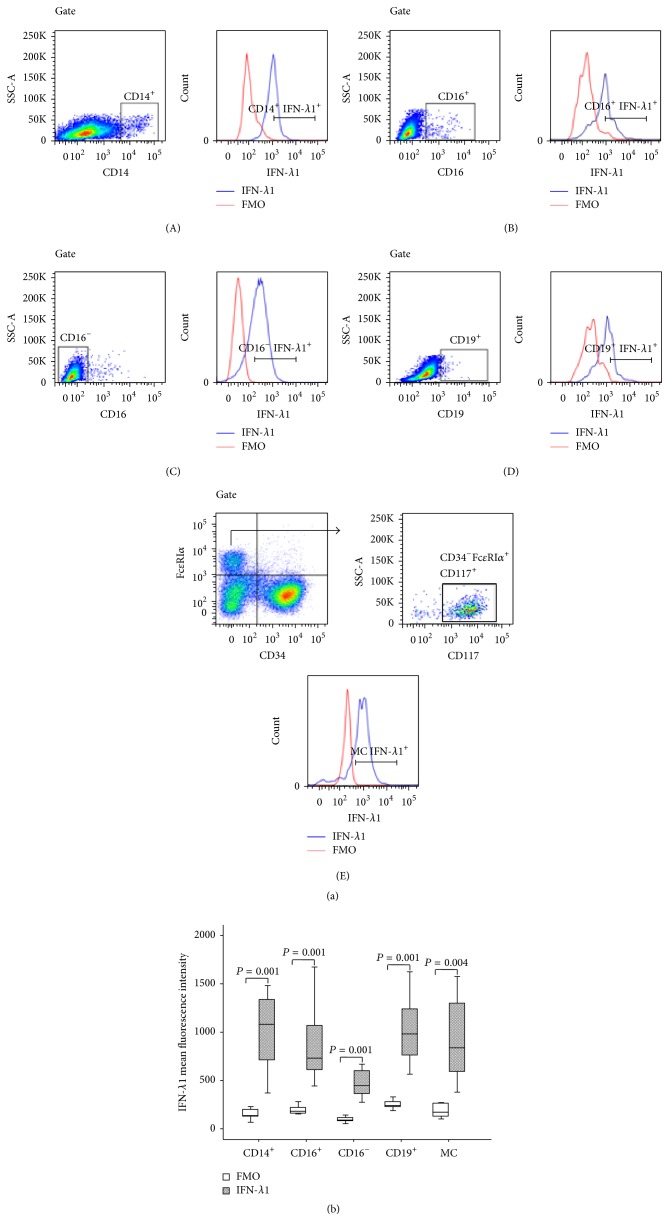
Flow cytometry analysis of the expression of interferon- (IFN-) *λ*1 in dispersed human skin cells. (a) showed a representative graph of the changes in the mean fluorescence intensity (MFI) of IFN-*λ*1 in CD14^+^ cells (macrophages), CD16^+^ cells (neutrophils), CD16^−^ (eosinophils), CD19^+^ cells (B cells), and CD34^−^CD117^+^Fc*ε*RI*α* ^+^ cells (mast cells) of skin tissue. Gate represents gating strategy. (b) represents a boxplot of the expression of IFN-*λ*1 in dispersed skin cells, which indicates the median (horizontal line), interquartile range, the largest, and smallest values other than outliers (whiskers) and the outliers (O). Each piece of data represented a group of minimum 6 subjects. *P* < 0.05 was taken as statistically significant.

Mildly upregulated expression of IFN-*λ*1 in CD8^+^, but not in CD4^+^, CD14^+^, CD16^+^, CD19^+^, CD16^−^, and CD123^+^HLA-DR^−^ blood cells of CSU, indicated that elevated plasma level of IFN-*λ*1 was unlikely generated from peripheral blood leukocytes. In order to further identify IFN-*λ*1 containing cells we investigated the various cell types in human skin tissues. The results showed that CD14^+^, CD16^+^, CD19^+^, and CD16^−^ cells and mast cells clearly expressed IFN-*λ*1 (Figure 3).

### 3.4. Immunohistochemical Analysis of IFN-*λ*1 Containing Cells in Skin

**Figure 4:**
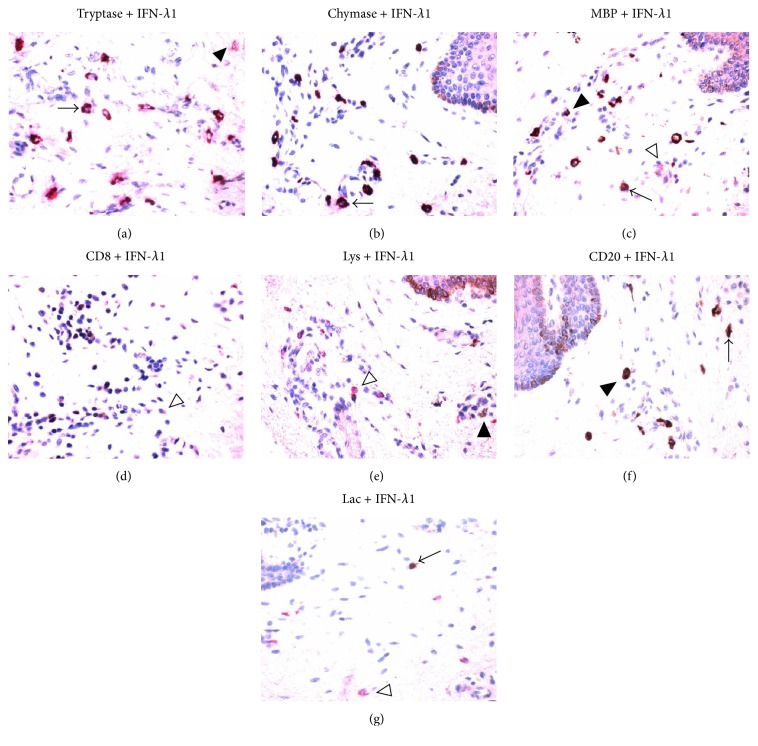
Double labeling immunohistochemical analysis of the expression of interferon- (IFN-) *λ*1 in human skin tissue. (a–g) showed representative graphs of the expression of IFN-*λ*1 (in brown, open arrow head) in (a) tryptase^+^ (in red, solid arrow head), (b) chymase^+^ (in red) mast cells, and (c) major basic protein (MBP)^+^ eosinophils (in red, solid arrow head); IFN-*λ*1 (in red, open arrow heads) in (d) CD8^+^ T cells (in brown), (e) lysozyme (Lys)^+^ macrophages (in brown, solid arrow head), (f) CD20^+^ B cells (in brown, solid arrow head), and (g) lactoferrin (Lac)^+^ neutrophils (in brown). Double labeled cells were in brown and red (arrows). Magnification was ×400. See Table 3.

**Figure 5:**
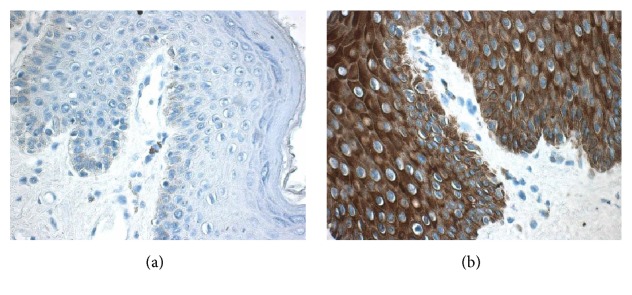
Immunohistochemical analysis of the expression of interferon- (IFN-) *λ*1 in human epidermis tissue. The tissue was fixed with Carnoy's solution and stained with (a) or without anti-IFN-*λ*1 antibody (negative control) (b). Positive stained cells were in brown. Magnification was ×400.

In order to confirm the above flow cytometric analysis result, double labeling immunohistochemical analysis technique was employed. The results revealed that IFN-*λ*1 containing cells were mainly located in the connective tissue of dermis (Figure 4). It was observed that approximately 3.8 tryptase^+^ mast cells, 58.7 chymase^+^ mast cells, 3.5 eosinophils, 1.6 B cells, 0.8 neutrophils, and 0.6 macrophages per mm^2^ skin tissue expressed IFN-*λ*1 (Table 3). However, T cells in the tissues examined did not express IFN-*λ*1 (data not shown). Using adult foreskin, it was found that human epidermis tissue highly expressed IFN-*λ*1 (Figure 5).

### 3.5. Induction of Inflammatory Cell Accumulation in Mouse Peritoneum by IFN-*λ*1

**Figure 6:**
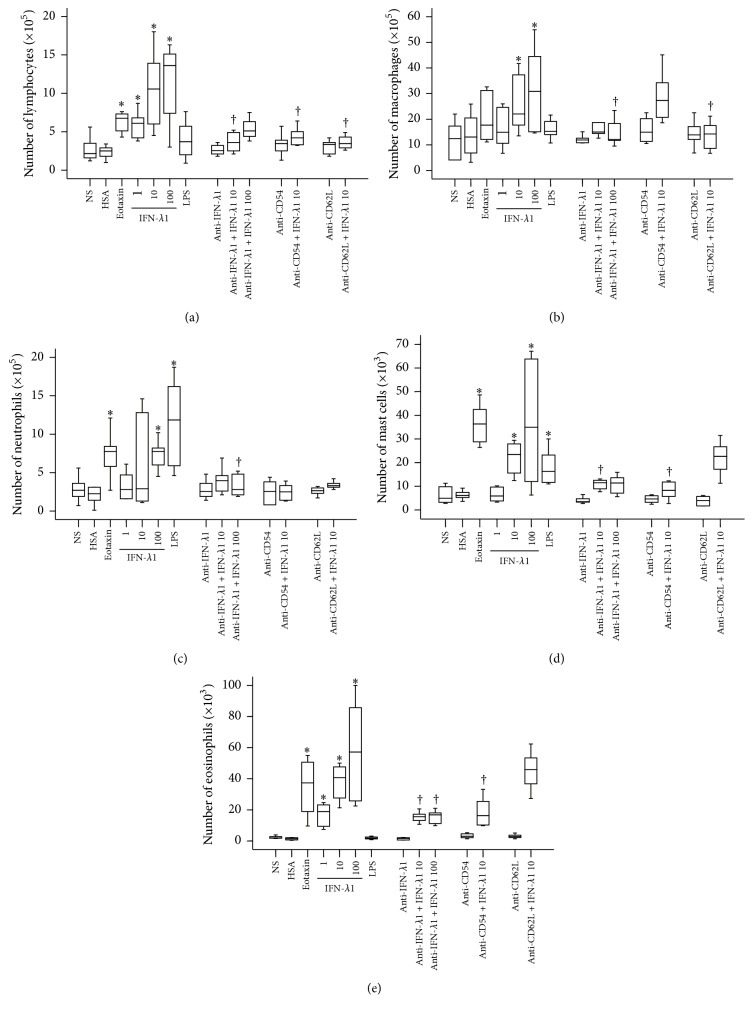
Interferon- (IFN-) *λ*1 induces inflammatory cell accumulation in mouse peritoneum. Mice were treated with IFN-*λ*1 (ng/mL) in the presence or absence of its specific antibody (3.0 *μ*g/mL), eotaxin (10 ng/mL), human serum albumin (HSA, 10 ng/mL), lipopolysaccharide (LPS, 1.0 *μ*g/mL), and normal saline (NS) for 6 h before their peritoneal lavage being collected for differential cell analysis. For certain mice, anti-CD54 antibody (anti-CD54, 1 mg·kg^−1^) and anti-CD62L antibody (anti-CD62L, 1 mg·kg^−1^) were injected via tail vein 30 min before IFN-*λ*1 being injected. Data were displayed as a boxplot, which indicates the median, interquartile range, the largest, and smallest values other than outliers (whiskers) and the outliers (O). Each piece of data represented a group of 6-7 animals. ^*∗*^ *P* < 0.05 compared with the corresponding NS group. ^†^ *P* < 0.05 compared with the corresponding stimulus alone group.

In order to examine the potential proinflammatory action of IFN-*λ*1, IFN-*λ*1 was injected into mouse peritoneum. The results showed that IFN-*λ*1 induced a dose-dependent increase in the number of lymphocytes (Figure 6(a)), macrophages (Figure 6(b)), neutrophils (Figure 6(c)), mast cells (Figure 6(d)), and eosinophils (Figure 6(e)) in the peritoneum of mice at 6 h following injection. In comparison, eotaxin provoked eosinophil, lymphocyte, mast cell, and neutrophil recruitment, whereas LPS elicited neutrophil and mast cell accumulation in the peritoneum of mice. Furthermore, anti-IFN-*λ*1 antibody blocked IFN-*λ*1-induced accumulation of lymphocytes, macrophages, neutrophils, mast cells, and eosinophils. While anti-ICAM-1 antibody inhibited IFN-*λ*1-induced accumulation of lymphocytes, neutrophils, mast cells, and eosinophils, anti-L-selectin antibody reduced IFN-*λ*1-induced recruitment of lymphocytes, macrophages, and neutrophils (Figure 6).

## 4. Discussion

We have observed for the first time that the level of IFN-*λ*1 was markedly higher in the plasma of the patients with CSU, but not with eczema. Since CSU is an immunological disease and IFN-*λ*1 is an immune active cytokine, which is associated with various diseases such as cancer, viral infection, and allergy, it is most likely that IFN-*λ*1 is involved in the pathogenesis of CSU. Lack of significant alteration of IFN-*λ*1 in plasma of patients with eczema and low concentration of IFN-*γ* in the plasma of CSU indicate that increased level of IFN-*λ*1 in CSU is specific.

To our surprise, the levels of IL-4 and TSLP were significantly lower in the plasma of CSU, which was very different from that observed in allergic disorders. It was previously shown that level of IL-4 was increased in the serum of allergic rhinitis [25] and allergic children [26], and TSLP were significantly increased in the serum of atopic dermatitis children [27]. Our findings provided further evidence that CSU is unlikely an allergic disease [28] though its IgE levels in blood appear to be elevated. The negative correlations between plasma IFN-*λ*1 and IL-4 and IL-10 and TSLP in the patients with CSU suggested that IFN-*λ*1 may not be released from the same cell source as the other three cytokines.

It is not too difficult to think that the increased level of IFN-*λ*1 was released from peripheral blood leukocytes as eosinophils [29] mononuclear cells [13], and monocyte-derived dendritic cells [10] were reported to secrete IFN-*λ*1. However, among the leukocytes examined, only CD8^+^ T cells expressed more IFN-*λ*1 in blood from CSU than blood from HC subjects, which can hardly explain the elevated plasma level of IFN-*λ*1. In order to find the potential source of IFN-*λ*1, we investigated expression of IFN-*λ*1 in human foreskin tissue. Because CD14^+^ and CD19^+^ cells and CD16^+^ and CD16^−^ granular cells and mast cells clearly expressed IFN-*λ*1 and there are a large number of mast cells, CD4^+^ T cells, monocytes, neutrophils, basophils [3], eosinophils [30], and B cells [31] in CSU skin tissue, we believe that the enhanced level of IFN-*λ*1 was most likely from these cells. It is worthy emphasizing that human epidermis tissue expresses huge amount of IFN-*λ*1, which could well be the source of plasma IFN-*λ*1 in CSU. Obviously, further study is required to clearly address the issue.

We also observed that IFN-*λ*1 was able to induce lymphocyte and neutrophil infiltration via an ICAM-1- and L-selectin-dependent mechanism. Since little information on cell migration induced by IFN-*λ* is available the reports that ICAM-1 is involved in each step of neutrophil extravasation [32] and CXC chemokine-induced neutrophil accumulation is dependent on neutrophil L-selectin [33] and that ICAM-1 mediates migration of Th1 and Th17 cells across human vascular endothelium [34] and L-selectin recruits Th1 cells in contact hypersensitivity of skin [35] may support our current observation. Similarly, the previous finding that monocyte migration to inflamed skin and lymph nodes is controlled by L-selectin [36] may help to understand our current observation that IFN-*λ*1 induced macrophage accumulation appeared to rely on activation of L-selectin. It was observed in the present study that IFN-*λ*1 induced recruitment of eosinophils and mast cells was through an ICAM-1-dependent manner. The findings that hapten-induced colonic eosinophilic inflammation is critically dependent on ICAM-1 [37] and that IL-4 provoked aggregation of human mast cells by promoting LFA-1/ICAM-1 adhesion molecules [38] may help to explain our current observation.

In conclusion, markedly elevated IFN-*λ*1 level in the plasma of patients with CSU suggests that IFN-*λ*1 plays a role in the pathogenesis of CSU through its ability in recruiting inflammatory cells. Tissue cells such as mast cells and macrophages rather than peripheral blood leukocytes were likely the source of plasma IFN-*λ*1. Blocking IFN-*λ*1 production may help to reduce the accumulation of inflammatory cells in the involved skin in CSU.

## Bulleted Statements

- Role of interferon- (IFN-) *λ*1 in innate immunity is recognized recently and regarded as a potent bioactive molecule. However, little is known about its role in chronic spontaneous urticaria (CSU).
- Markedly elevated IFN-*λ*1 level in CSU plasma suggests that IFN-*λ*1 is involved in CSU.
- The ability in recruiting inflammatory cells suggests that IFN-*λ*1 plays a role in the pathogenesis of CSU.
- IFN-*λ*1 could be generated from tissue inflammatory cells.

## Figures and Tables

**Table 1:** 

	HC	CSU	Eczema
	(*n* = 32)	(*n* = 23)	(*n* = 18)
Age (yr)	35 (18–60)	45 (13–68)	37 (14–69)
Female/male	17/15	11/12	11/7
Median age at onset (yr)	na	27 (8–56)	30 (12–60)
Median disease duration (yr)	na	2.5 (0.5–20)	3 (1–10)
Blood taken time after the first symptom of the latest attack (h)	na	11 (6–20)	8 (2–15)
Number of positive skin pricks for dust mite	0	5	4
Number of positive skin pricks for *Artemisia*	0	3	2
Number of positive skin pricks for ragweed	0	2	1
Number of positive skin pricks for cat fur	0	2	1
Number of positive skin pricks for dog fur	0	1	2

Median (range) data are shown for the number of subjects indicated.

na: not applicable.

**Table 2:** The spearman's rho/correlation coefficient between the plasma level of IFN-*λ*1, IL-4, IL-10, and TSLP. ^*∗*^ *P* < 0.05.

CSU				
	IFN-*λ*1	IL-4	IL-10	TSLP
IFN-*λ*1	1	−0.848^*∗*^	−0.925^*∗*^	−0.975^*∗*^
IL-4	−0.848^*∗*^	1	0.886^*∗*^	0.875^*∗*^
IL-10	−0.925^*∗*^	0.886^*∗*^	1	0.964^*∗*^
TSLP	−0.975^*∗*^	0.875^*∗*^	0.964^*∗*^	1

**Table 3:** The number of IFN-*λ*1 expressing cells (double positive cells)/mm^2^ and the percentage of IFN-*λ*1 expressing cells in each cell type examined. Data shown are the median (range) value from 6 different donors. Positively stained cells in each section were counted for more than 30 fields (magnification ×400).

Cell type	Number of double positive cells/mm^2^	% of double positive cells in each cell type
Try^+^MC	3.8 (2.1–10.6)	81.1 (61.5–96.2)
Chy^+^MC	58.7 (27.0–120.4)	93.9 (93.7–97.5)
Eosinophils	3.5 (1.9–7.6)	26.2 (17.8–52.6)
Macrophages	0.6 (0.4–1.9)	52.8 (34.8–87.7)
CD8^+^T cells	0.0 (0.0–0.0)	0.0 (0.0–0.0)
B cells	1.6 (1.1–6.6)	92.9 (62.1–93.2)
Neutrophils	0.8 (0.3–1.2)	20.1 (15.2–30.6)
